# Supplementary material for: Is It Possible to Notice the Unmet Non-Medical Needs among Cancer Patients? Application of the Needs Evaluation Questionnaire in Men with Lung Cancer
Source: Curr Oncol. 2023 Mar 20;30(3):3484–93. doi: 10.3390/curroncol30030264 (PMC10047714; doi:10.3390/curroncol30030264)
Supplement: Supplementary file 1 [file curroncol-30-00264-s001.zip › consent form.pdf]

ZGODA NA UCZSTNICTWO W BADANIU

Świadomie i dobrowolnie wyrażam zgodę na uczestnictwo w badaniu pt.: „Walidacja standaryzowanego kwestionariusza ankietowego NEQ (Needs Evaluation Questionnaire) w polskiej populacji pacjentów z chorobą nowotworową, analiza jakościowa i ilościowa potrzeb pozamedycznych pacjentów".

Wyrażam zgodę na wykorzystanie uzyskanych informacji do celów naukowych i ewentualnej publikacji.

Udział w badaniu jest anonimowy. Żadne dane osobowe nie zostaną ujawnione.
Ochrona danych osobowych zgodnie z Ustawą z dnia 10 maja 2018 r. o ochronie danych osobowych.

....
Data Podpis badanego

....
Data Podpis osoby informującej
