# Supplementary material for: Is It Possible to Notice the Unmet Non-Medical Needs among Cancer Patients? Application of the Needs Evaluation Questionnaire in Men with Lung Cancer
Source: Curr Oncol. 2023 Mar 20;30(3):3484–93. doi: 10.3390/curroncol30030264 (PMC10047714; doi:10.3390/curroncol30030264)
Supplement: Supplementary file 1 [file curroncol-30-00264-s001.zip › NEQ Polish version.pdf]

KWESTIONARIUSZ Nr ……..

1. Potrzebuję więcej informacji na temat mojej choroby
   - ☐ Tak
   - ☐ Nie
2. Potrzebuję więcej informacji na temat mojego stanu zdrowia w przyszłości
   - ☐ Tak
   - ☐ Nie
3. Potrzebuję więcej informacji dotyczących wykonywanych badań
   - ☐ Tak
   - ☐ Nie
4. Potrzebuję więcej informacji na temat mojego leczenia
   - ☐ Tak
   - ☐ Nie
5. Chciałabym/chciałbym w większym stopniu uczestniczyć w podejmowaniu decyzji dotyczących mojego leczenia
   - ☐ Tak
   - ☐ Nie
6. Chciałabym/chciałbym, aby lekarze i pielęgniarki przekazywali mi informacje bardziej zrozumiałym językiem/w bardziej zrozumiały sposób
   - ☐ Tak
   - ☐ Nie
7. Chciałabym/chciałbym, aby lekarze byli ze mną bardziej szczerzy
   - ☐ Tak
   - ☐ Nie
8. Potrzebuję bliższego kontaktu z lekarzem
   - ☐ Tak
   - ☐ Nie
9. Chciałabym/chciałbym, aby moje objawy (np. ból, nudności, bezsenność) były lepiej łagodzone
   - ☐ Tak
   - ☐ Nie
10. Potrzebuję więcej pomocy przy czynnościach takich jak jedzenie, ubieranie się, korzystanie z toalety
    - ☐ Tak
    - ☐ Nie
11. Potrzebuję większego poszanowania mojej intymności/prywatności
    - ☐ Tak
    - ☐ Nie
12. Chciałabym/chciałbym, aby pielęgniarki okazywały mi więcej uwagi/zainteresowania
    - ☐ Tak
    - ☐ Nie
13. Chciałabym/chciałbym, aby lekarze umieli rozpraszać moje wątpliwości
    - ☐ Tak
    - ☐ Nie

14. Chciałabym/chciałbym, aby w szpitalu były lepsze warunki (np. stan łazienek, jakość posiłków)
    - Tak
    - Nie
15. Potrzebuję więcej informacji dotyczących należnych mi świadczeń socjalnych związanych z moją chorobą
    - Tak
    - Nie
16. Potrzebuję pomocy finansowej
    - Tak
    - Nie
17. Chciałabym/chciałbym mieć możliwość spotkania/rozmowy z psychologiem
    - Tak
    - Nie
18. Chciałabym/chciałbym mieć możliwość spotkania/rozmowy z księdzem
    - Tak
    - Nie
19. Chciałabym/chciałbym mieć możliwość spotkania/rozmowy z osobami, które mają taką samą chorobę
    - Tak
    - Nie
20. Chciałabym/chciałbym, aby moi bliscy bardziej mnie wspierali
    - Tak
    - Nie
21. Chciałabym/chciałbym czuć się bardziej potrzebna/y mojej rodzinie
    - Tak
    - Nie
22. Chciałabym/chciałbym czuć się mniej samotna/y
    - Tak
    - Nie
23. Chciałabym/chciałbym, aby ludzie okazywali mi mniej współczucia/litości
    - Tak
    - Nie

METRYCZKA Nr ………

1. Płeć:
- ☐ kobieta
- ☐ mężczyzna

2. Wiek: …………lat

3. Wykształcenie:
- ☐ podstawowe
- ☐ średnie/zawodowe
- ☐ wyższe

4. Miejsce zamieszkania
- ☐ miasto, jakie ….…………….
- ☐ wieś

5. Aktywność zawodowa
- ☐ uczeń/student
- ☐ nadal pracuję zawodowo
- ☐ w chwili obecnej L4 lub zasiłek rehabilitacyjny
- ☐ bezrobotny
- ☐ emeryt
- ☐ rencista

6. Stan cywilny
- ☐ mężatka/żonaty lub w stałym związku
- ☐ mój związek zakończył się w trakcie lub w związku z chorobą
- ☐ panna/kawaler
- ☐ rozwiedziona/rozwiedziony
- ☐ wdowa/wdowiec

7. Mieszkam
- ☐ tylko ze współmałżonkiem lub partnerem/partnerką
- ☐ ze współmałżonkiem lub partnerem/partnerką i z dzieckiem/dziećmi
- ☐ tylko z dzieckiem/dziećmi
- ☐ z innym członkiem rodziny
- ☐ samotnie

8. Czy ktoś z Pani/Pana najbliższej rodziny lub przyjaciół jest lekarzem?
   - ☐ tak
   - ☐ nie

9. Rozpoznanie choroby:……………………………………………

10. Przybliżona data rozpoznania choroby:…………………………
(miesiąc/rok)
