# Supplementary material for: Is It Possible to Notice the Unmet Non-Medical Needs among Cancer Patients? Application of the Needs Evaluation Questionnaire in Men with Lung Cancer
Source: Curr Oncol. 2023 Mar 20;30(3):3484–93. doi: 10.3390/curroncol30030264 (PMC10047714; doi:10.3390/curroncol30030264)
Supplement: Supplementary file 1 [file curroncol-30-00264-s001.zip › Title of Table S1.pdf]

Table S1. The correlations between unmet non-medical needs and demographic's factors.
